# Enhanced long-term memory and increased mushroom body plasticity in *Heliconius* butterflies

**DOI:** 10.1016/j.isci.2024.108949

**Published:** 2024-01-18

**Authors:** Fletcher J. Young, Amaia Alcalde Anton, Lina Melo-Flórez, Antoine Couto, Jessica Foley, Monica Monllor, W. Owen McMillan, Stephen H. Montgomery

**Affiliations:** 1 Department of Zoology, University of Cambridge, Downing Street, Cambridge CB2 3EJ, UK; 2 Smithsonian Tropical Research Institute, Gamboa, Panama; 3 School of Biological Science, University of Bristol, 24 Tyndall Avenue, Bristol BS8 1TQ, UK

**Keywords:** Entomology, Neuroscience, Evolutionary biology

## Abstract

*Heliconius* butterflies exhibit expanded mushroom bodies, a key brain region for learning and memory in insects, and a novel foraging strategy unique among Lepidoptera – traplining for pollen. We tested visual long-term memory across six *Heliconius* and outgroup Heliconiini species. *Heliconius* species exhibited greater fidelity to learned colors after eight days without reinforcement, with further evidence of recall at 13 days. We also measured the plastic response of the mushroom body calyces over this time period, finding substantial post-eclosion expansion and synaptic pruning in the calyx of *Heliconius erato*, but not in the outgroup Heliconiini *Dryas iulia*. In *Heliconius erato*, visual associative learning experience specifically was associated with a greater retention of synapses and recall accuracy was positively correlated with synapse number. These results suggest that increases in the size of specific brain regions and changes in their plastic response to experience may coevolve to support novel behaviors.

## Highlights

• *Heliconius* butterflies have enhanced visual long-term memory compared to outgroups
• *Heliconius* recall learned color associations up to 13 days without reinforcement
• Mushroom bodies of *H. erato* show greater developmental plasticity than *Dryas iulia*
• In *H. erato*, learning experience is linked to mushroom body synapse number

## Graphical abstract

**Figure:**
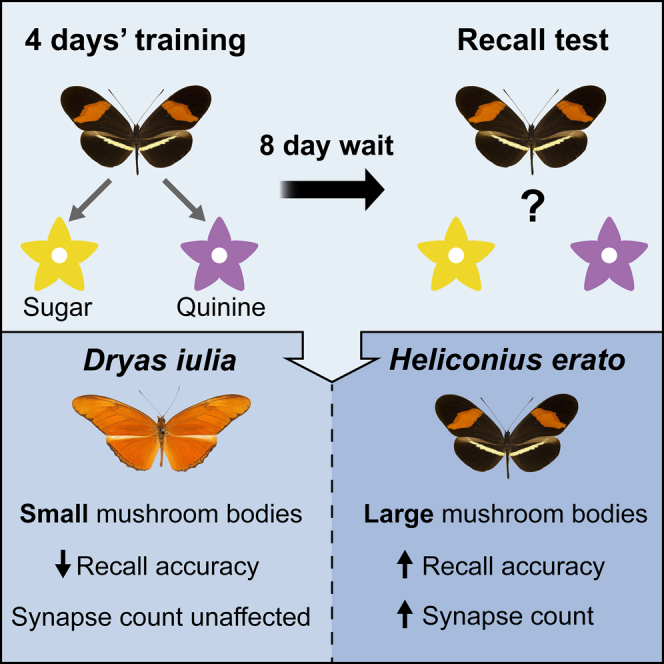

## Introduction

Animals vary markedly in cognitive ability, both within and between species, yet the neural traits determining these differences are only partly understood, particularly in an evolutionary context.^1^ A major strand of research has focused on linking measures of “intelligence” to brain size^2^^,^^3^^,^^4^ and several studies have found correlations between brain size and certain cognitive abilities.^5^^,^^6^^,^^7^ However, attempts to link brain size to cognition have been also critiqued, particularly in an interspecific context.^8^^,^^9^^,^^10^ Whole brain size can ignore variation in brain composition and connectivity^11^^,^^12^^,^^13^^,^^14^ and nonlinear scaling relationships between brains regions.^15^ Comparative behavioral studies may also fail to consider important ecological differences between species, casting doubt over the biological relevance of the assessed cognitive tasks.^9^^,^^16^ Many of these issues can be avoided by conducting studies within species,^17^^,^^18^^,^^19^ or among closely related species,^20^ focusing on linking specific neural traits and cognitive abilities.^9^ In insects, the mushroom bodies have been a focus of investigation as a higher-order integrative center for learning and memory.^21^^,^^22^ The mushroom bodies have undergone expansion in several lineages including Hymenoptera,^12^ cockroaches,^23^ herbivorous scarab beetles,^24^ and *Heliconius* butterflies.^25^^,^^26^^,^^27^ Notably, the behavioral functions of the mushroom bodies vary substantially between insect groups, particularly in the relative importance of visual and olfactory modalities.^22^

Synaptic plasticity, which involves the reorganization and differential strengthening and pruning of synaptic connections between neurons, also plays a key role in learning and memory.^28^^,^^29^ As such, neural plasticity and neural expansion may act as two, non-independent, axes underpinning cognitive evolution. The insect mushroom body exhibits a high degree of post-eclosion volumetric expansion,^18^^,^^30^^,^^31^ which often reflects changes in the number and density of microglomeruli,^17^^,^^32^^,^^33^^,^^34^^,^^35^^,^^36^ synaptic complexes formed by connections between sensory projection neurons and Kenyon cells, the intrinsic neurons of the mushroom body.^37^ The combined effects of high interspecific variability in size and composition, and high intraspecific plasticity, make the mushroom bodies an excellent model for investigating the neural underpinnings of cognitive evolution. However, few studies have investigated the links between variation in mushroom body size and/or plasticity and cognitive differences between closely related species.^38^

**Figure 1:**
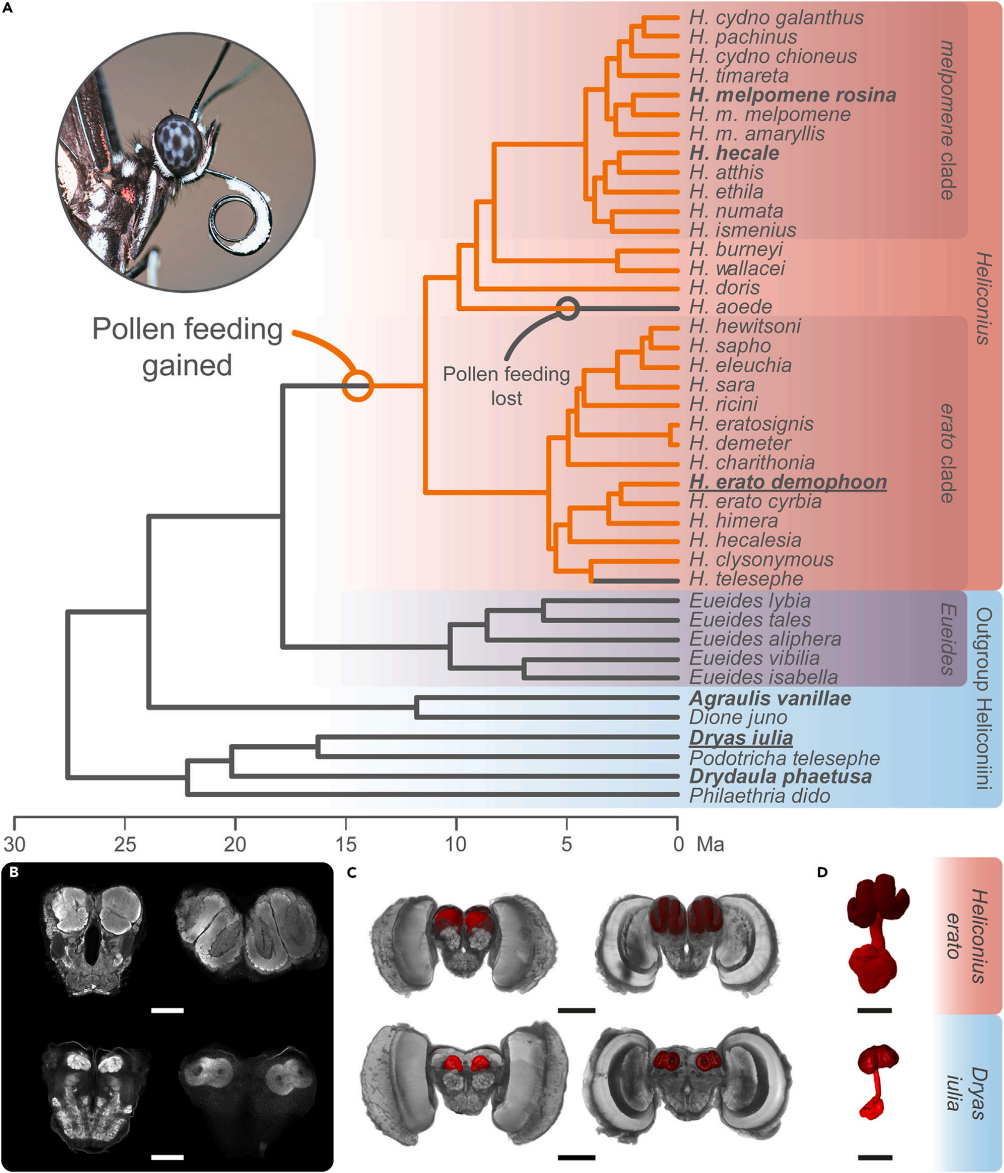
Mushroom body expansion and pollen feeding in *Heliconius* (A) Distribution of pollen feeding in Heliconiini butterflies with dated phylogeny adapted from Couto et al. (2023). Long-term memory assays were conducted using the bolded species, while brains were sampled from species also underlined. (B‒D) Mushroom bodies in *Heliconius erato* and *Dryas iulia*. (B) Confocal scans showing the anterior (left) and posterior (right) of the central brain (scale bars = 250 μm). (C) 3D-reconstructions of the whole brain showing anterior (left) and posterior (right) with the mushroom bodies (red) (scale bars = 500 μm). (D) Isolated 3D-reconstructions of the mushroom body showing the calyx (dark red) and peduncles and lobes (light red) scale bars = 250 μm).

The Neotropical butterfly genus *Heliconius*, and their Heliconiini allies, are an emerging system for investigating cognitive evolution.^39^ *Heliconius* have markedly expanded mushroom bodies relative to other Heliconiini^25^^,^^26^^,^^27^ (Figure 1), with this increase primarily explained by increased neuron number and volumes of calyx receiving innervations from visual, rather than olfactory, processing centers.^25^ This expansion event occurred relatively recently (∼12–18 Ma)^40^ and coincides with the dietary innovation of adult pollen feeding and associated derived foraging behaviors^41^^,^^42^ (Figure 1). *Heliconius* are able to form long-term memories of spatial information over large scales,^43^ and establish “traplines” along stable pollen foraging routes.^44^^,^^45^^,^^46^ Pollen feeding provides an adult source of amino acids, and has co-evolved alongside a major increase in lifespan and reproductive longevity.^47^ This extended adult period likely increases the benefit of long-term memories. Given their established role in long-term memory^32^^,^^33^^,^^48^ and visual learning^17^^,^^49^^,^^50^^,^^51^ in other insects, changes in long-term memory may be intimately linked to mushroom body expansion in *Heliconius* butterflies. Indeed, initial comparative data have shown that *Heliconius erato* outperform the Heliconiini *Dryas iulia* in their ability to learn and recall complex visual cues and maintain long-term memories of learned color associations.^25^

Here, we extend previous data by testing visual long-term memory across additional species to build a dataset of three *Heliconius* (*Heliconius erato*, *Heliconius melpomene* and *Heliconius hecale*) and three outgroup Heliconiini (*Dryas iulia*, *Agraulis vanillae* and *Dryadula phaetusa*). We use this to test for consistent, superior memory fidelity across the *Heliconius* genus, providing considerably greater basis for generalization of effects. We subsequently investigate the neural basis of this behavioral difference, examining calyx volume, synapse density—inferred from an active zone marker—and the number of Kenyon cells in *H. erato* and *D. iulia* that participated in the long-term memory assay, in conjunction with age-matched controls, and freshly eclosed butterflies. We quantify neural plasticity in these individuals, and test for neural correlates of learning experience.

## Results and discussion

### *Heliconius* show consistently superior visual long-term memory relative to outgroup Heliconiini

**Figure 2:**
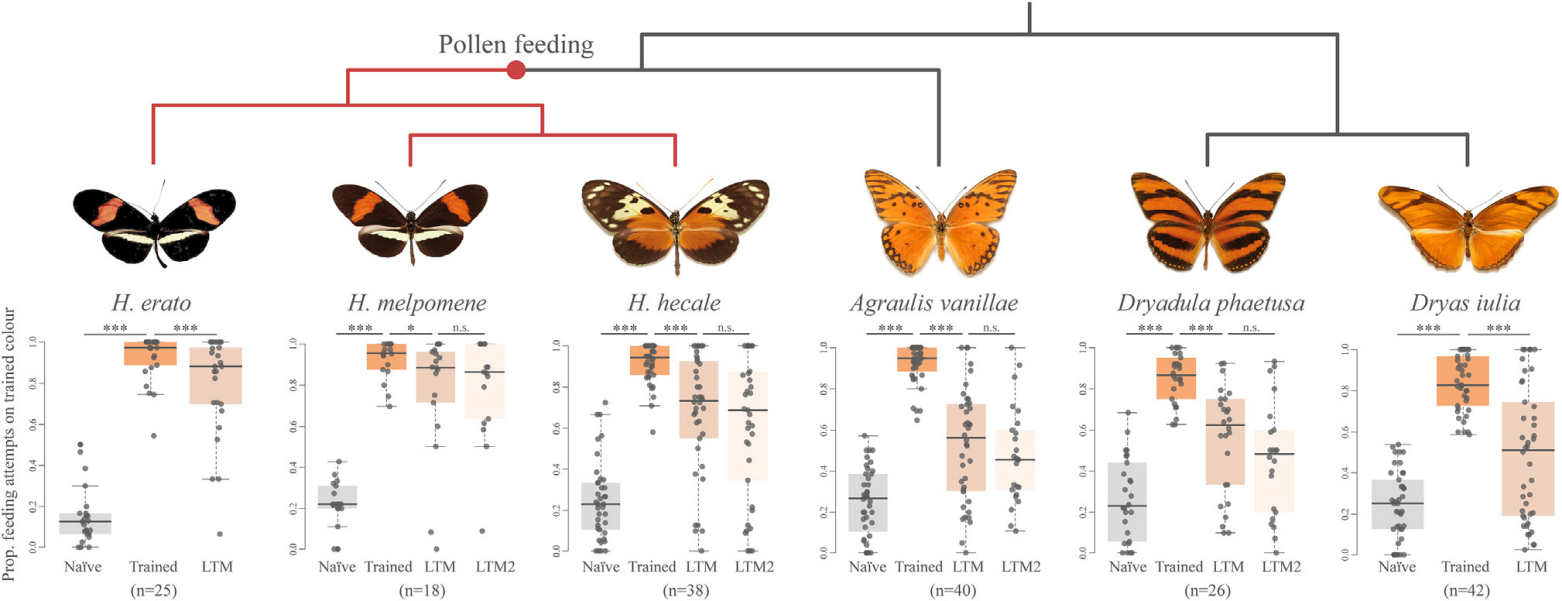
*Heliconius* show superior visual long-term memory relative to outgroup Heliconiini Long-term memory accuracy in six Heliconiini species in a two-color preference assay. The boxes encompass the two middle quartiles, with central line showing median. Whiskers extend to the furthest data point within 1.5 times the interquartile range. Trained = 16-h recall performance after four days training. LTM = recall performance after eight days deprived of the learned color stimuli by being fed solely on neutral (white) feeders; LTM2 = recall performance after an additional four days on white feeders. n.s. = no statistically significant difference; ∗ = p < 0.05; ∗∗∗ = p < 0.001 with P-values calculated using a z-test and corrected with the Šidák correction. Butterfly images from Warren et al. (2023).^53^

We trained butterflies to associate a sugar-protein reward with either yellow or purple feeders over four days and then tested their recall accuracy after 16 h (STAR methods). *Heliconius* exhibited slightly higher accuracy over non-*Heliconius* individuals in an initial recall test (Figure 2; Table S3; z ratio = −2.240, p = 0.048), but all six species successfully learned the color-food association (Figure 2; Table S4). To test the stability of these learnt associations over a longer period of time, butterflies were then deprived of the learned color cues and fed solely on white feeders for eight days. After this period, we conducted subsequent preference trials using purple and yellow feeders with *Heliconius* individuals exhibiting significantly greater fidelity to the previously learned colors (Figure 2; Table S5; z ratio = −5.807, p < 0.0001). Furthermore, while all species exhibited a decline in accuracy over the eight days between the initial trained test and the long-term memory test (Figure 2; Table S4), this drop-off was significantly higher for non-*Heliconius* individuals than in *Heliconius* (χ^2^ = 5.309, d.f. = 1, p = 0.0212). We further assayed the recall abilities of *H. melpomene*, *H. hecale*, *A. vanilla*, and *D. phaetusa* after an additional four days deprived of the learned color cues (a total of 13 days without reinforcement), again finding higher accuracy in *Heliconius* individuals (Figure 2; z ratio = −3.731, d.f. = inf, p < 0.001). At this point, the color preferences of *A. vanillae* and *D. phaetusa* were not different from random (Table S6), suggesting a loss of the learnt association. In contrast, *H. melpomene* maintained their learned preference during this period, while *H. hecale* also exhibited a marginally nonsignificant bias for the learned color (Table S6). To our knowledge, this period of 13 days represents the longest example of the persistence of a learned association without reinforcement in an insect, ahead of an 11-day period tested in the honeybee.^52^

Importantly, our results extend previous data showing that *Heliconius erato* has more stable long term memories than *Dryas iulia*.^25^ By including additional *Heliconius* species and outgroup species, we sampled major clades within *Heliconius* and across the Heliconiini, to confirm consistent shifts between *Heliconius* and other Heliconiini. These data strongly suggest that *Heliconius* as whole possess an enhanced visual long-term memory relative to other Heliconiini. This difference is consistent with mushroom body expansion in *Heliconius* being associated with an improved visual long-term memory, further supported by the dominant role of increased visual processing in *Heliconius* mushroom body expansion.^25^ This behavioral change may have been driven by the cognitive demands of traplining for pollen in the context of increased individual longevities.^41^ The role of the mushroom bodies in the formation and maintenance of olfactory long-term memories is well established.^32^^,^^48^^,^^54^ However, the present results add to evidence that for at least certain groups of insects, including many Hymenoptera,^36^ the mushroom bodies also play a significant role in visual long-term memory.^21^^,^^22^ In contrast to the significant clade effects we find in our long term memory assays, comparisons across a similar sample of Heliconiini species did not find *Heliconius* to be superior at learning shape cues^55^ or reversal learning of color cues.^56^ When coupled with our present results, these findings suggest that mushroom body expansion in *Heliconius* has not led to an overall improvement in general cognition, but rather enhancement in specific, ecologically relevant cognitive tasks.

### Mushroom bodies of *Dryas iulia* and *Heliconius erato* vary in their response to learning experience

**Figure 3:**
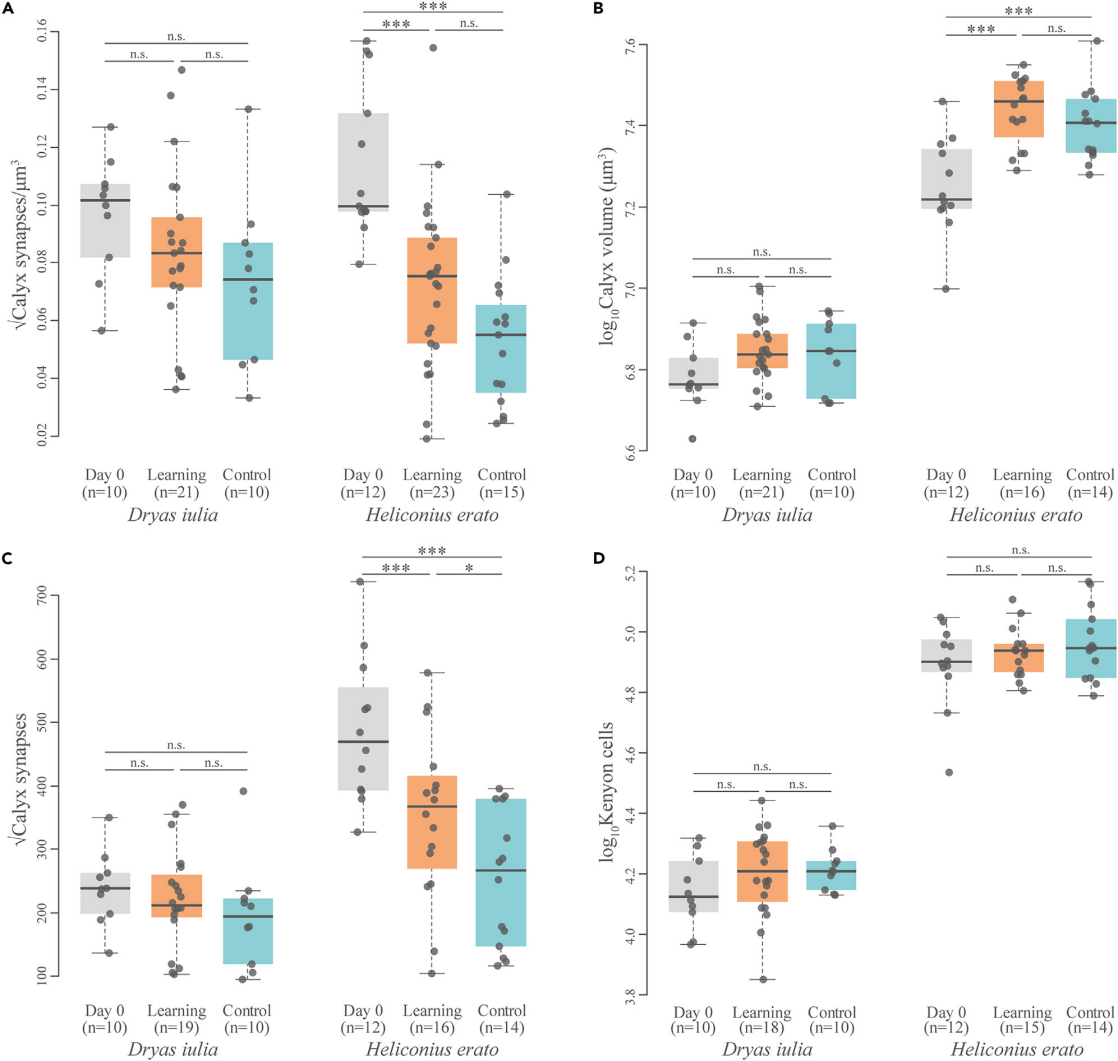
Mushroom bodies of *Dryas iulia* and *Heliconius* erato vary in their response to learning experience Variation in (A) synapse density, (B) volume, (C) and total synapse number of the mushroom body calyx, and (D) total Kenyon cell number, between three treatment groups, Day 0, Learning and age-matched Controls, in *Dryas iulia* and *Heliconius erato*. The boxes encompass the two middle quartiles, with central line showing median. Whiskers extend to the furthest data point within 1.5 times the interquartile range. All values correspond to a single hemisphere of the brain. n.s. = no statistically significant difference; ∗ = p < 0.05; ∗∗ = p < 0.01; ∗∗∗ = p < 0.001 with P-values calculated using a t-test and corrected with the Šidák correction.

We further explored the neural underpinnings of this cognitive shift in *Heliconius* at the cellular and synaptic level by using immunohistochemistry to measure several traits in the mushroom body calyx of *Heliconius erato* and *Dryas iulia* that had completed the long-term memory assay (Learning group). These individuals were compared with age-matched individuals from a “non-learning” environment (Control group) and freshly eclosed butterflies (Day 0 group). The “non-learning” group experienced the same experimental set up as the learning group, but both colors were equally reinforced and punished, preventing a conditioned preference (Figure S3; Table S7). Overall, when comparing Day 0 butterflies to either the Control or Learning groups, the mushroom bodies of *Heliconius erato* showed considerably more plasticity than *D. iulia* (Figure 3; Tables S10–S12). In *Heliconius erato*, the Learning and Control group individuals had a lower synapse density and fewer total synapses in the calyx, but increased calyx volumes, relative to Day 0 individuals (Figures 3A–3C; Table S11). In contrast, for *D. iulia*, although similar trends were observed, neither synapse density, calyx volume nor synapse number varied significantly between groups (Figure 3; Table S11). Age-associated increases in calyx volume, and decreases in synapse density, similar to those we observe in *H. erato* have also been described in a number of Hymenoptera including bumblebees,^17^^,^^57^ honeybees,^34^^,^^58^ ants^35^^,^^38^ and paper wasps.^59^^,^^60^ The decrease in synapse number with age observed in *H. erato* also parallels observations in honeybees^34^^,^^58^^,^^61^ and desert ants.^35^ This “pruning” of synapses, which is also present in vertebrates, is a recognized method of refining neural connectivity involving the selective elimination of axonal branches and increasing the strength of remaining synaptic connections.^62^

Notably, individual *H. erato* in the Learning group had a significantly higher number of synapses in the calyx than the Control group individuals (Figure 3C; Table S11), which have similar synapse counts to *D. iulia* despite having much larger calyces (Figure 3; Table S11). This suggests a higher maintenance of synapses in the Learning group in *H. erato* and more extensive synaptic pruning in the Control group. The experience of the Learning and Control groups was identical, except for the reinforcement of color cues during the four-day training period, a relatively modest environmental difference. These differences persisted for eight days after exposure to colored feeders, suggesting that learning-associated synaptic connections in the calyx are being maintained for considerable amounts of time after exposure. Increased density or number of mushroom body synapses has previously been linked to visual^17^ and olfactory^32^^,^^33^ learning and long-term memory in Hymenoptera, and these results extend those findings to visual learning and memory in *Heliconius*. Interestingly, in *Drosophila*, synaptic reorganization of the mushroom body appears to be essential for only certain associative learning tasks, being necessary for aversive, but not appetitive, associative odor learning.^63^

*Heliconius erato* did not show an increase in calyx volume in response to associative color learning specifically (Figure 3B), suggesting learning and memory of the color cues was achieved solely through synaptic reorganization. Contrasting with our findings, visual learning has been linked with calyx growth in honeybees^17^ and the butterfly *Pieris* rapae.^18^ In honeybees^32^ and leaf-cutting ants,^33^ however, olfactory learning has been linked to increased synapse density without expansion of the calyx. In addition, we extend previous reports of an absence of adult neurogenesis in Heliconiini,^64^ by showing that learning experience does not promote an increase in Kenyon cell number (Figure 3; Table S11), as observed in some hemimetabolous insects.^65^^,^^66^ Differences in synapse count between *Heliconius erato* groups therefore appear to be a result of changes in the number of synapses per Kenyon cell (Table S12). Day 0 individuals had significantly more synapses per Kenyon cell than Control individuals, but not the Learning group (Table S12). Together with the lack of neurogenesis in honeybees,^67^ the present findings suggest that, for some insect groups, learnt associations are supported primarily through plasticity in synapse strength and number without adult neurogenesis, despite its importance in some vertebrate brain regions.^68^

### Recall accuracy in *Heliconius erato*, but not *Dryas iulia*, is associated with increased synapse density and number in the mushroom body calyx

**Figure 4:**
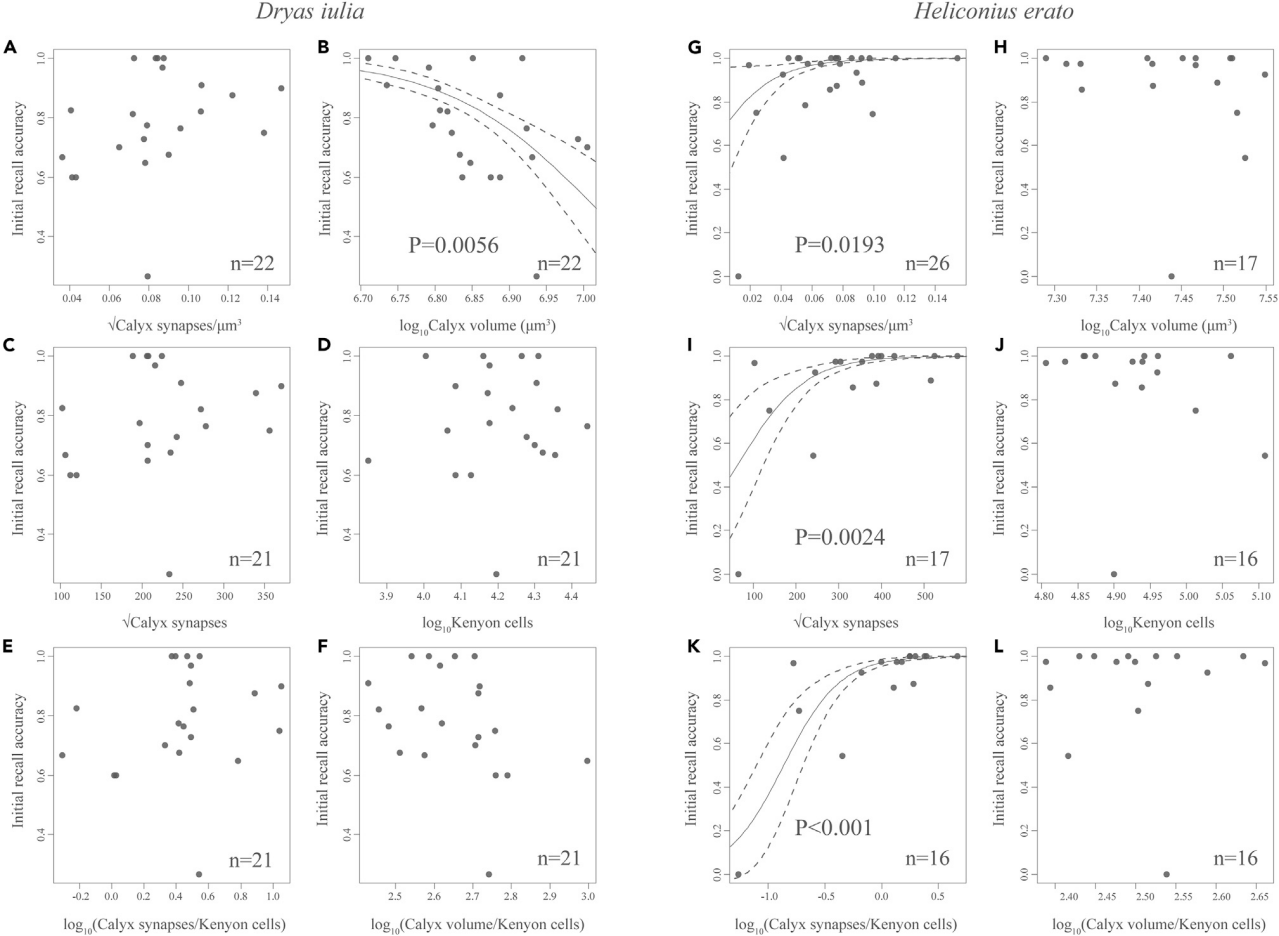
Recall accuracy in *Heliconius erato*, but not *Dryas iulia*, is associated with increased synapse density and number in the mushroom body calyx (A–L) Relationship between 16-h recall accuracy of a learned food-color association in a two-color preference test and several neuroanatomical measurements in the mushroom body calyx for (A‒F) *Dryas iulia* and (G‒L) *Heliconius erato*. All values correspond to a single hemisphere of the brain. Regression lines, derived from generalized linear mixed models, with standard errors, are shown only when the trait was a significant predictor of performance, assessed using a chi-square test.

We further tested for specific neural correlates of within-species variation in recall performance in both the initial recall (16 h removed from the color cues) and long-term recall tests (eight days removed). For *Heliconius erato*, performance in the initial recall test was positively correlated with calyx synapse density (χ^2^ = 5.473, d.f. = 1, p = 0.0193) and number (χ^2^ = 9.199, d.f. = 1, p = 0.0024), and the ratio of synapses to Kenyon cells (χ^2^ = 12.852, d.f. = 1, p < 0.001) (Figures 4G, 4I, and 4K; Table S13). Together with our prior finding of learning experience being associated with an increased calyx synapse count in *Heliconius erato*, this strongly suggests that the synaptic connections in the calyx between Kenyon cells and projection neurons from primary visual neuropils are playing a key role in visual learning and memory. This mirrors data from honeybees where a similarly positive correlation is reported between synapse density in the mushroom body collar, an area of the calyx receiving solely visual input, and visual memory.^17^ Unlike *H. erato*, initial recall accuracy performance in *D. iulia* was not correlated with calyx synapse density or number (Figures 4A, 4C, and 4E; Table S13), but was, surprisingly, *negatively* correlated with calyx volume (Figure 4B; Table S13). This is suggestive of an altered relationship between mushroom plasticity and the consolidation of the learned food-color association between these two species. Neither species showed any correlation between performance in the long-term recall test and measured traits in the calyx (Figure S4). However, when a clear outlier individual (E43) is removed, positive relationships with synapse count (χ^2^ = 9.263, d.f. = 1, p = 0.002) and the ratio of synapses to Kenyon cells (χ^2^ = 10.918, d.f. = 1, p = 0.001) were recovered in *H. erato* (Figure S5). No other data point has this significant an effect on the result (Table S14), while the result is highly stable once it is removed (Table S15). This may suggest the associations detected above also persist to the 8-day recall trial. Regardless, the above results suggest an important role for synaptic reorganization in the mushroom body calyx in the formation of visual associative memories in *Heliconius*.

### Behavioral innovation and the coevolution of mushroom body volume and plasticity

Our data evidence a distinct shift in visual long-term memory ability in *Heliconius* coinciding with expansion of the mushroom body and changes in its developmental plasticity. These changes in memory stability likely co-occur with the emergence of trapline foraging, which depends on the long-term memory of resource locations and provides the selective advantage for increased memory longevity.^41^ Comparative neuroanatomical studies have tended to focus on variation in the volume of the brain, or specific brain regions.^9^ However, our findings indicate that the evolution of neural plasticity is an important axis to consider, and that the size of specific brain structures and their plasticity may coevolve to support behavioral adaptations. Finally, we provide an important step toward revealing the evolutionary changes in neural connectivity, and information processing and storage, that occur in expanded learning and memory circuits. With these results, *Heliconius* butterflies offer an example of how structural changes in the brain can support a distinct shift in visual memory, providing an important case study toward understanding the evolution of cognition.

### Limitations of the study

*Eueides*, the sister genus to *Heliconius*, exhibit mushroom bodies intermediate in size between *Heliconius* and other Heliconiini^25^ and would make an excellent inclusion in the present experiment. However, owing to their smaller size and shorter proboscises, *Eueides* spp. do not engage with the artificial feeders used in this experiment as readily as other Heliconiini genera and consequently tend to not perform well in these types of behavioral assays. We note, however, that *Eueides* mushroom bodies are intermediate in size on a log scale only, and much smaller in absolute volume compared with *Heliconius*.^25^ In addition, our experiment does include *D. phaetusa*, which have mushroom bodies of a size comparable to *Eueides*,^25^ and perform in line with other outgroup Heliconiini. We also note that, unlike in Hymenoptera,^69^ the subdivisions of the calyx receiving visual and olfactory input are not easily visually discernible in the Heliconiini. We therefore used our synapse density measurements to estimate synapse numbers for the entire calyx rather than regions receiving visual input specifically, assuming a homogeneity of synapse density across the calyx. Given the visual nature of the learning task in this study, synaptic reorganization could be expected to be heightened in visual regions of the calyx, relative to other regions. Therefore, this method of approximation has the potential to dampen the apparent magnitudes of the group differences that we detect. Finally, these results could be strengthened by collecting neural plasticity data for the other four Heliconiini species included in the behavioral assays to confirm whether the differences we detect between *Heliconius erato* and *Dryas iulia* are repeated clade-wide.

## STAR★Methods

### Key resources table

**Table:** 

REAGENT or RESOURCE	SOURCE	IDENTIFIER
**Antibodies**		

Mouse anti-SYNORF1: 3C11	DSHB	RRID:AB_2315424
Rabbit anti-horseradish peroxidase, P-7899	Sigma-Aldrich	RRID:AB_261181
Cy2 goat anti-mouse IgG: 115-225-146	Jackson ImmunoResearch	RRID: AB_2307343
Cy3 goat anti-rabbit IgG: 111-165-144	Jackson ImmunoResearch	RRID: AB_2338006

**Chemicals, peptides, and recombinant proteins**		

DAPI (4′,6-diamidino-2-phenylindole)	ThermoFisher Scientific	Catalog number: D1306
HEPES	Sigma-Aldrich	CAS number: 7365-45-9
Critical Care Formula	Vetark	N/A
Tris	ThermoFisher Scientific	Catalog number: 17926
Normal goat serum	ThermoFisher Scientific	RRID:AB_2532166
Phosphate buffered saline	ThermoFisher Scientific	Catalog number: BR0014G
Triton X-100	SigmaAldrich	CAS number: 9036-19-5

**Deposited data**		

Behavioral data	This paper	Mendeley Data: https://doi.org/10.17632/fg2fvpzsfr.1
Neuroanatomical data	This paper	https://doi.org/10.17632/fg2fvpzsfr.1

**Software and algorithms**		

Amira 3D v 2021.2	ThermoFisher Scientific	N/A
ImageJ	Schneider et al.^70^	https://imagej.nih.gov/ij/
Modular Image Analysis plugin for ImageJ	Cross^71^	https://imagej.net/plugins/modularimageanalysis/
Stardist plugin for ImageJ	Schmidt et al.^72^	https://imagej.net/plugins/stardist
R v 4.1.0	R Core Team	https://www.r-project.org/
glmmTMB v 1.1.2.3 package for R	Brooks et al.^73^	https://cran.r-project.org/web/packages/glmmTMB/index.html
lme4 v 1.1–21 package for R	Bates et al.^74^	https://cran.r-project.org/web/packages/lme4/index.html
emmeans v 1.7.0 package for R	Lenth^75^	https://cran.r-project.org/web/packages/emmeans/index.html
DHARMa v 0.4.4 package for R	Hartig^76^	https://cran.r-project.org/web/packages/DHARMa/index.html
smatr v 3.4–8 package for R	Warton et al.^77^	https://cran.r-project.org/web/packages/smatr/index.html
R code for statistical analyses	This paper	Mendeley Data: https://doi.org/10.17632/fg2fvpzsfr.1

### Resource availability

#### Lead contact

Further information and requests for resources and reagents should be directed to and will be fulfilled by the lead contact, Fletcher James Young (fletcherjyoung@gmail.com).

#### Materials availability

This study did not generate new unique reagents.

#### Data and code availability

• Behavioral and neuroanatomical data have been deposited at Mendeley Data and are publicly available as of the date of publication. The DOI is listed in the key resources table. Raw confocal microscope images collected in this study will be shared by the lead contact upon request.
• All original code has been deposited at Mendeley Data and is publicly available as of the date of publication. The DOI is listed in the key resources table.
• Any additional information required to reanalyze the data reported in this paper is available from the lead contact upon request.

### Experimental model and study participant details

The behavioral assays in this study used six Heliconiini species.

• *Agraulis vanillae*
• *Dryadula Phaetusa*
• *Dryas iulia*
• *Heliconius erato*
• *Heliconius hecale*
• *Heliconius melpomene*

All experiments used freshly eclosed, adult, captive-reared butterflies with no prior experience. After eclosion, butterflies were kept in a mesh pop-up and transferred to pre-training the next morning. Individuals were, therefore, 1-day-old upon beginning the behavioral assay. For *Heliconius erato* and *Dryas iulia*, Learning group individuals were dissected the evening after completing the 8-day recall test and were therefore aged to at least 17 days. Control group individuals were aged-matched to the Learning group. Day-0 individuals were dissected the evening of the day they eclosed.

Butterfly stock populations were established with locally-caught, wild butterflies and maintained at the insectaries at the Smithsonian Tropical Research Institute in Gamboa, Panama. Stock butterflies were kept in 2 × 2 × 3m mesh cages in ambient conditions with natural light. Larvae were reared in mesh pop-ups and were provided with fresh leaves daily. *H. erato*, *Dryas iulia*, *Dryadula phaetusa* and *Agraulis vanillae* were reared on *P. biflora*, *H. melpomene* on *P. triloba*, and *H. hecale* on *P. vitifolia*. Training and testing of butterflies was conducted in 2 × 2 × 3m mesh cages in ambient conditions under natural light. A single *Palicourea elata*, with all flowers removed, was placed in the rear right corner of these cages as a roosting site. *H. erato* and *Dryas iulia* individuals were further dissected for the neuroanatomical investigations.

Sex was randomly distributed for all species. We initially included sex effects in these models, but found that it had a significant effect on calyx volume and Kenyon cells number in *H. erato* only (Table S9), consistent with previous findings of marginally larger mushroom bodies in female *Heliconius*, but not outgroup Heliconiini.^25^ Sex was therefore included as a random effect when testing for differences in calyx volume and Kenyon cell number only using the function *glmmTMB* from the package *glmmTMB* v 1.1.2.3 for R.^78^ *H. erato* and *Dryas iulia* individuals were randomly assigned to the Learning, Control and Day 0 treatment groups.

### Method details

#### Long-term memory assay

Long-term memory (LTM) experiments, using color cues, were carried out on captive-reared butterflies between January and April 2019 in Gamboa, Panama. The experiments used two colors, purple and yellow, colors chosen based on previous experiments using *H. erato* which showed that neither color was particularly attractive.^73^ The experiments used five-pointed, star-shaped, artificial feeders made from colored foam, 3 cm in diameter, with a centrally placed 0.5 mL Eppendorf tube that could be filled with liquid.

The day after eclosion, individuals were transferred to a pre-training cage, where they were fed solely with white artificial feeders containing a sugar-protein solution (20% sugar, 5% Vertark Critical Care Formula, 75% water, w/v) for two days (from 08:00 to 12:00) to familiarise them with the use of artificial feeders. After pre-training, butterflies were introduced to a testing cage to determine initial feeding preferences between purple and yellow. Testing cages contained 12 purple and 12 yellow feeders arranged randomly in a 4 X 6 grid, with 6.5 cm between feeders on each side. To ensure that butterflies responded exclusively to visual cues, feeders in the testing cages were empty. Preference testing lasted for 4 h from 08:00 to 12:00 and was filmed from above using a GoPro Hero 5 camera mounted on a tripod. Butterflies were individually numbered on their wings for identification using a permanent marker. The film was then reviewed to count the number of feeding attempts per individual on each color, with up to 40 attempts recorded per individual. A feeding attempt was only counted if the butterfly landed on the feeder and probed it with its proboscis.

Butterflies were then trained to associate a food reward with their non-favoured color, based on the results of their initial preference test. For butterflies that initially preferred purple, the training cage contained yellow feeders containing a sugar-protein solution, and purple feeders containing a saturated quinine solution, an aversive stimulus. The opposite arrangement was employed for individuals that initially preferred yellow. This training period lasted for four full days. After training, butterfly preferences were re-tested, following the same protocol as the initial preference test, to verify that individuals had indeed acquired the colour-food association. After the trained preference test, butterflies were placed for eight days in a cage identical to the pre-training cage, containing only white feeders filled with a sugar-protein solution. The deprivation of color stimuli for this period allowed for testing the long-term memory retention of the colour-food association acquired during the training period, and ensured that long-term memory was being tested rather than short-term or mid-term memory.^79^ A period of eight days was chosen because *Heliconius* are known to maintain their foraging routes over periods ranging from weeks to months,^45^^,^^46^^,^^80^^,^^81^ during which time a pollen resource could be unproductive for several days due to competition or damage, but ultimately rewarding over the long term. Butterflies were then subject to a third preference test to determine if the learned preference was maintained, following the same protocol as the initial preference test. *H. melpomene*, *H. hecale*, *Dryadula phaetusa* and *Agraulis vanilla* individuals were also subject to an additional extended long-term memory test by placing them in a cage with only white for a further four days before a final preference test.

#### Kenyon cell and synapse counting and calyx measurement

##### Treatment groups

Neuroanatomical measurements were taken for *Dryas iulia* and *Heliconius* erato from three treatment groups – Day 0, Learning and Control. Day 0 butterflies comprised individuals that were dissected the evening of their day of emergence. These butterflies were kept in a small, mesh pop-up cage until dissection and had no foraging or free-flight experience. The Learning Group comprised *Dryas iulia* and *Heliconius erato* individuals that participated in the long-term memory experiment and were dissected the day they finished (Learning Group). The Control Group consisted of individuals aged-matched to the Learning group reared in a “non-learning” environment. Control individuals were acclimatised to the use of artificial feeders and then tested for naive color preference following the same protocol used for the Learning Group. These butterflies were then introduced to a “training” cage for four days, which contained an even number of purple and yellow feeders, presented in random spatial arrangement. Unlike the Learning group, where feeder color was consistently associated with either a food reward or quinine punishment, half of the feeders for each color were filled with the rewarding sugar-protein mixture, and half with the aversive quinine solution. Each color, therefore, was as equally rewarding as punishing. After four days in this environment, color preference was tested again following the protocol established for the long-term memory experiment. The Control butterflies were then introduced to a cage with white feeders for eight days, replicating the long-term memory waiting period of the Learning group. Finally, after eight days feeding on white feeders, Control butterflies were exposed to empty purple and yellow feeders from 08:00 to 12:00 on their final morning, mirroring the last preference test of the Learning Group, before being dissected in the evening.

##### Brain dissection, fixing and training

All brains were dissected and fixed at the Smithsonian Tropical Research Institute in Gamboa, Panama, following established protocols.^26^^,^^82^ Butterflies were decapitated using scissors and the head was submerged under HEPES-buffered saline (HBS; 150 mM NaCl; 5 mM KCL; 5 mM CaCL_2_; 25 mM sucrose; 10 mM HEPES; pH 7.4). A small aperture was cut into the head cuticle between the eyes to improve permeation of the fixative. The brain was fixed *in situ* for 16–20 h at room temperature under gentle agitation in zinc-formaldehyde solution (ZnFA; 0.25% [18.4 mM] ZnCl_2_; 0.788% [135 mM] NaCl; 1.2% [35 mM] sucrose; 1% formaldehyde). After fixing, the whole brain was dissected out under HBS using a scalpel and forceps, placed into 80% methanol, 20% dimethyl sulfoxide (DMSO) under agitation for 2 h and then transferred to 100% methanol for long-term storage at −20°C.

Brains were subject to three immunohistochemical stains.^25^ Anti-synapsin was used to mark active zones of synapses in the calyx^17^^,^^32^^,^^61^; this approach has previously been used as a proxy for synapse density/number and provides comparable results when equivalent estimates of microglomeruli density/number are made when double stained with a post-synaptic marker, anti-phalloidin.^25^ Due to the need for long-term storage of the brains during fieldwork, we use a protocol that includes methanol, and therefore prohibits successful anti-phalloidin staining.^25^ We note that variation in staining intensity could be caused by differential up/down-regulation at synapses with different degrees of activity. Our density estimates may therefore partially capture variation in synapse active zone activity as well as variation in number. We also used DAPI to identify nuclei in Kenyon cell bodies,^82^^,^^83^ along with HRP (anti-horseradish peroxidase) to label neuron membranes to confirm that counted nuclei were neuronal.^84^ Brains were stained in batches of eight that included individuals from all treatment groups to avoid the possibility of batch effects skewing results. Prior to staining, brains were first rehydrated in a decreasing methanol series (90%, 70%, 50%, 30%, 0% in 0.1 M Tris buffer, pH 7.4) for 10 min each. Because quantification of the Kenyon cells and synapses requires imaging the brain at 63× magnification, it was necessary to section the brains so that the calyx tissue would be within the working distance of the objective lens of the microscope. Brains were embedded in 5% agarose which was cut into a rectangular prism. The agarose block was cut along a corner so that individual slices could be correctly orientated later during mounting. The embedded brain was submerged in 0.1 M Tris buffer and sliced horizontally into 80 μm sections using a Leica VT1000 S vibrating blade microtome.

After sectioning, brain slices were blocked in PBSd-NGS (1% DMSO, 5% normal goat serum (NGS) diluted in 0.1 M phosphate-buffered saline (PBS; 7.4 pH)) for 2 h. A mono-clonal antibody targeting synapsin (mouse anti-SYNORF1: 3C11, DSHB, RRID:AB_2315424; [1:30]) and HRP (Rabbit anti-horseradish peroxidase, P-7899, Sigma-Aldrich, RRID:AB_261181; [1:5000]) were diluted in PBSd-NGS, then applied at a 1:30 dilution in PBSd-NGS and kept for three days at 4°C under low agitation. Samples were then washed in PBS (3 × 2 h) before applying the Cy2-conjugated secondary antibody (Cy2 goat anti-mouse IgG: 115-225-146, Jackson ImmunoResearch, RRID: AB_2307343; [1:100]) and Cy3-conjugating secondary antibody (Cy3 goat anti-rabbit IgG: 111-165-144, Jackson ImmunoResearch, RRID: AB_2338006; [1:200]), in PBSd-NGS. Samples were then kept at 4°C under low agitation for a further three days. Samples were then rinsed in PBS (3 × 2 h). In preparation for the DAPI stain, samples were washed in 0.2% Triton in distilled H_2_O for 10 min. DAPI was applied 1:1000 in 0.2% Triton and H_2_O under agitation for 30 min at room temperature. After, samples were permeabilised in 0.2% Triton and H_2_O for 10 min and then in 0.2% Triton and PBS (4 × 10 min) and then placed overnight in 60% glycerol in PBS. Brain sections were then mounted in 80% glycerol on slides under a coverslip sealed with nail polish and stored in the dark. Due to the delicate nature of the 80 μm slices, samples from some individuals suffered physical damage during staining and mounting meaning that their total calyx volume, and synapse and Kenyon cell counts could not be reconstructed.

##### Confocal microscopy and image processing

All brains were imaged using a laser-scanning confocal microscope (Upright Leica SP5, Leica Microsystem, Mannheim, Germany), at a resolution of 1024 × 1024 pixels. Mushroom body calyces were scanned using 10× dry objective (0.4 NA), with a mechanical *z*-step of 1 μm, with each brain section scanned individually. Kenyon cells and synapses were imaged using the 63× objective (1.3 NA) under a glycerol immersion, with a mechanical *z*-step of 1 μm. For each individual, five regions of mushroom body calyx and Kenyon cell cluster were chosen at random for scanning. Cy2 was excited with the Argon laser at 488 nm. The solid-state lasers were used to excite DAPI at 405 nm and Cy3 at 561 nm. Wavelengths were scanned sequentially and received on photomultiplier tubes.

Brain images were processed using ImageJ v 1.53n and Amira 3D 2021.2. Calyx volumes were reconstructed using Amira, with each brain section segmented separately. For each stack, every third or fourth image was manually segmented by highlighting the region covered by the calyx and then interpolated across the *z*-dimension (Figures S1A and S1B). The *measure statistics* function was used to extract volumes (in μm^3^) for each section, which were later added together for the total calyx volume and a correction factor of 1.85 was applied. Synapse densities were estimated using ImageJ.^70^ Following protocols established in Couto et al. (2023), the *3D Objects Counter* function was used to automatically count 3D objects within five 50 × 50 × 15μm boxes (Figures S1C and S1D). For each scan, the brightness threshold was adjusted manually so that only distinct objects were counted. To reduce noise, objects smaller than 10 voxels were not counted. Note, unlike Hymenoptera, the visual and olfactory regions of the Heliconiini calyx lack clear morphological boundaries, meaning we were unable to reliably place boxes specifically in visual or olfactory calyx. The total number of synapses in the calyx was then estimated by calculating the average synapse density across the five scans and multiplying it by the total calyx volume, assuming homogeneity of synapse densities across the calyx. Kenyon cell cluster volumes and total Kenyon cell numbers were determined in a similar manner. Kenyon cell density was estimated from five randomly selected 25 × 25 × 15μm boxes in the DAPI-stained cell cluster, using the HRP stain as an aid to verify cell bodies were neuronal (Figures S1E and S1F). Cell numbers within each box were automatically counted using the *Modular Image Analysis* (MIA) and *Stardist* plugins in ImageJ.^64^^,^^71^^,^^72^ *Stardist* detects objects with star-convex shape priors and can be used for detecting cells. Total cell counts were then estimated by multiplying the average density by the total volume of the cell cluster. We note, Kenyon cells numbers for the Day 0 and Control groups are also reported in Alcalde et al. (2023).

### Quantification and statistical analysis

Performance in the long-term memory trials was analyzed with generalised linear mixed models (GLMM) using the *glmer* function from the *lme4* package v 1.1–21 in R v 4.1.0.^74^ All models used a binomial distribution with purple and yellow feeding choices as dependent variables and individual-ID was included as a random effect. Post-hoc comparisons among relevant pairs of species, clades, or trials were made by obtaining the estimated marginal means using the package *emmeans* v 1.7.0^75^ and were corrected for multiple comparisons using Šidák correction. To test for interspecific differences in performance, species was included as a fixed effect. Differences between *Heliconius* and outgroup Heliconiini were tested by including membership in the *Heliconius* genus and trial as fixed effects with species as a random effect. To test for potential differences between *Heliconius* and non-*Heliconius* in the drop in performance between the initial preference test and the long-term memory test, an interaction between *Heliconius*-membership and trial was included. To account for overdispersion, an observation-level random effect was included. Significant deviation from random color preference during the initial preference test and the second long-term memory test was assessed using a null generalised linear mixed model. Diagnostics for all models were assessed using the package *DHARMa* v 0.4.4.^76^ Individuals that exhibited less than 50% accuracy during the initial trained preference test, and therefore did not appear to have learned the food-colour association, were removed from the dataset. In total, 9 individuals were so removed from the dataset: 1 out of 26 *H erato* individuals were removed, in addition to 2 of 20 *H melpomene*, 2 of 40 *H hecale*, 2 out of 42 *Agraulis vanillae*, 1 of 42 *Dryas iulia*, 1 of 27 *Dryadula phaetusa*. A generalised linear model treating species as a fixed effect did not show significant variation between species in the proportion of individuals scoring less than 50% during the first training test (χ^2^ = 1.514, d.f. = 5, p = 0.911).

*Heliconius erato* and *Dryas iulia* synapse densities and counts were first square-root transformed, and calyx volume and Kenyon cell counts were log_10_ transformed to better fit a normal distribution. We then ran a series of generalised linear models (GLMs) with a Gaussian distribution using the *glm* function in R v 4.1.0 testing for differences in these neuroanatomical traits between groups within species and between species within groups. Species and group, and their interaction, were, thus, included as effects. For these analyses, we removed two individuals from the Learning group, one from each species, whose accuracy was less than 50% during the initial recall test, on the basis that those individuals had not acquired a learned association with the trained color. All post-hoc comparisons were made by obtaining the estimated marginal means using the package *emmeans* v 1.7.0^75^ and were corrected for multiple comparisons using the Šidák correction. We further tested for variation in the scaling relationships between Kenyon cell number and number of synapses in the calyx, and Kenyon cell number and calyx volume, using the *sma* function in the R package *smatr* v 3.4–8.^77^ This analysis allows for the detection of shifts in elevation in the scaling relationship between two traits. The “robust” option was set to true for these analyses and multiple comparisons were corrected for.^85^ Finally, we tested whether specific neural traits correlate with recall performance in the initial recall test and long-term memory test. For each species, we used *glmmTMB* to run a series of binomial GLMMs, with the trait of interest as a fixed effect and ID as a random effect. These analyses were repeated for both the initial recall test and the long-term recall test. During our analyses, we identified a single *Heliconius erato* (E43) that exhibited highly unusual behavior, making 16 out of 16 correct feeding attempts in the initial recall test, but only 2 out of 28 correct attempts in the long-term recall test. To test whether this individual had an outsized statistical effect on our analyses, we therefore re-ran these models excluding each individual one at a time. This analysis showed that statistical effect of removing E43 from the dataset was not matched by any other individual (Table S14). We then further tested whether removing this individual from the dataset produces stable results by re-running the models excluding both E43 and the remaining individuals one at a time, finding that the results recovered when removing E43 are robust (Table S15). On this basis, we include an analysis of the dataset excluding E43 in the Supplementary Material (Figure S5; Tables S14 and S15).
