# Supplementary material for: Enhanced long-term memory and increased mushroom body plasticity in Heliconius butterflies
Source: iScience. 2024 Jan 18;27(2):108949. doi: 10.1016/j.isci.2024.108949 (PMC10864207; doi:10.1016/j.isci.2024.108949)
Supplement: Document S1. Figures S1–S5 and Tables S1–S15 [file mmc1.pdf]

iScience, Volume *27*

Supplemental information

Enhanced long-term memory and increased mushroom body plasticity in *Heliconius* butterflies

Fletcher J. Young, Amaia Alcalde Anton, Lina Melo-Flórez, Antoine Couto, Jessica Foley, Monica Monllor, W. Owen McMillan, and Stephen H. Montgomery

Enhanced long-term memory and increased mushroom body plasticity in *Heliconius* butterflies - Supplementary Figures and Tables

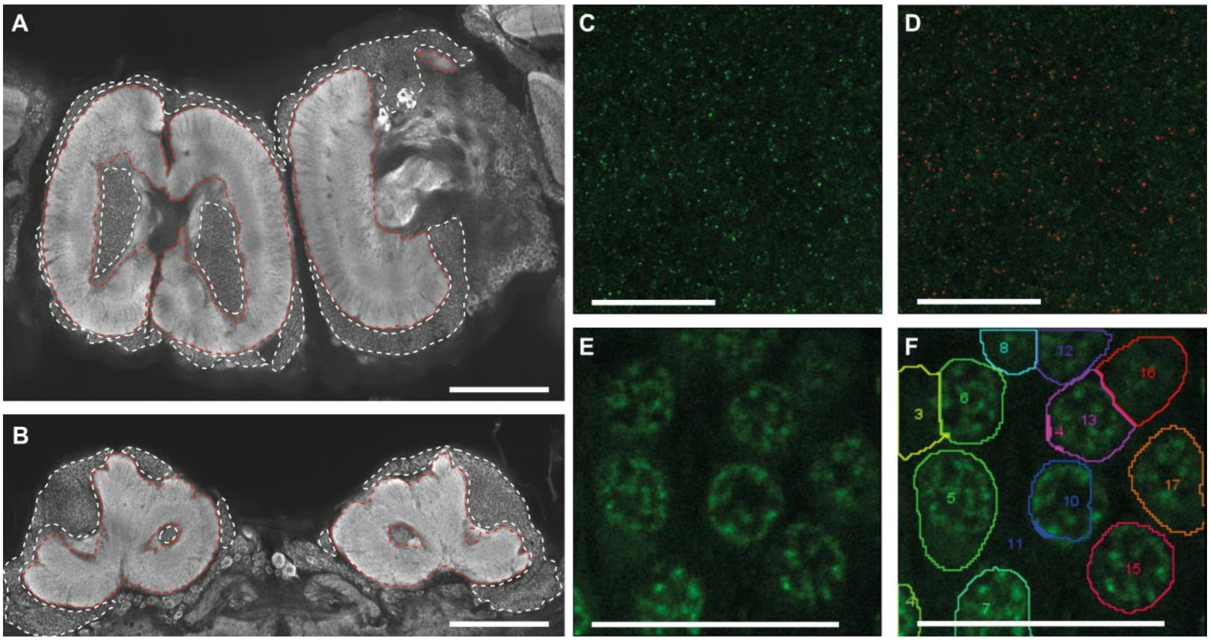

Figure S1. Laser-scanning confocal microscope images showing examples of immunohistochemistry staining of the mushroom bodies and automated synapse and cell counting techniques described in STAR methods. **(A)** HRP staining of the mushroom bodies in *Heliconius erato* and **(B)** *Dryas iulia* from the Learning group. White dashed line shows the calyx. Red dashed line shows the Kenyon cell cluster. Scale bar represents 200 μm. **(C)** Calyx synapses in *Heliconius erato* revealed through anti-synapsin staining, which are **(D)** automatically counted using *3D Object Counter* in ImageJ. **(E)** Kenyon cell nuclei in *Heliconius erato* revealed through DAPI staining which are **(F)** automatically counted using *Modular Image Analysis* and *Stardist* in ImageJ. **(C)-(F)** Scale bars represents 20 μm.

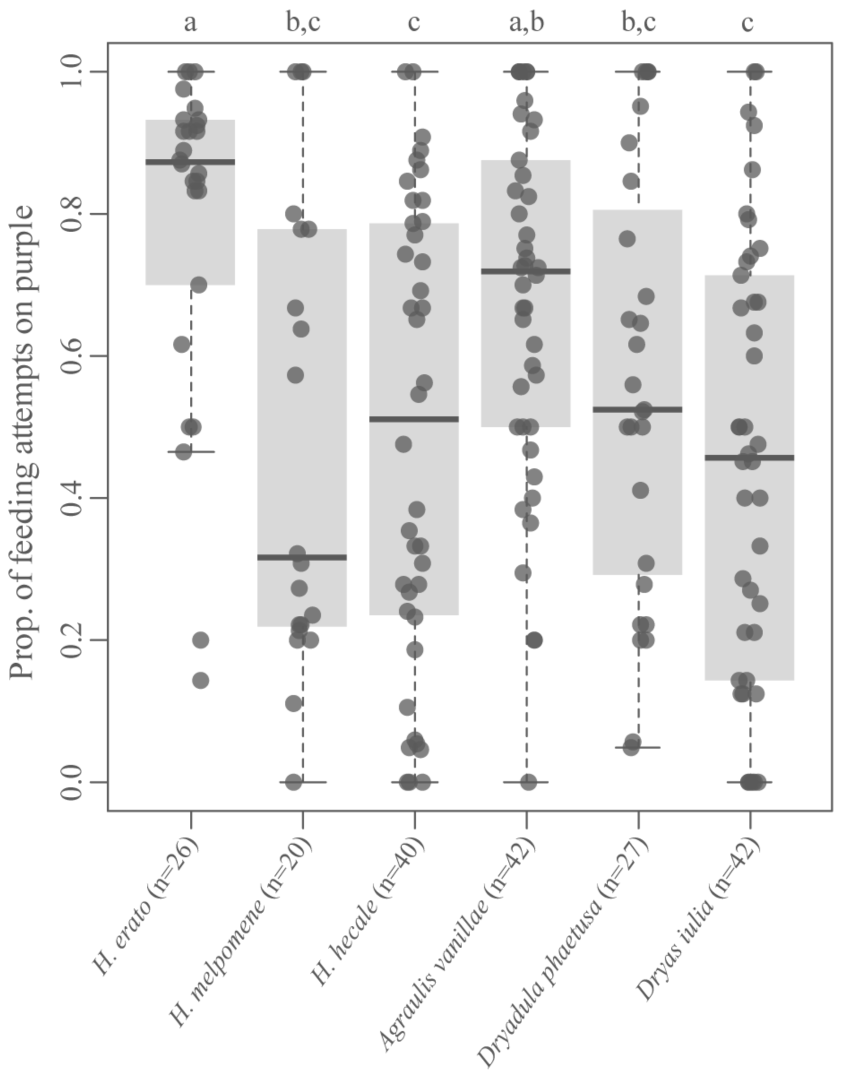

Figure S2. Significant variation (χ^2=33.968, d.f.=5, P<0.0001) in Naïve colour preference between purple and yellow for three *Heliconius* three non-*Heliconius* Heliconiini, related to Figure 2. The boxes encompass the two middle quartiles, with central line showing median. Whiskers extend to the furthest data point within 1.5 times the interquartile range. Notation at the top shows significantly different pairwise comparisons based on Table S1.

Table S1. Pairwise comparisons between species in naïve preference between purple and yellow feeders, related to Figures 2 and S2. Bottom left shows z-ratio and top right the associated P-value, corrected for multiple comparisons using the Šidák correction. Asterisks indicate significant pairwise differences. * = P<0.05, ** = P<0.01, *** = P<0.001.

	H. erato	*H. melpomene*	*H. hecale*	*A. vanillae*	*D. phaetusa*	*D. iulia*
H. erato		0.0050**	0.0001***	0.737	0.045*	<0.0001***
H. melpomene	3.585**		1.000	0.225	1.000	1.000
H. hecale	4.439***	0.189		0.020*	0.967	1.000
Agraulis vanillae	1.721	-2.391	-3.213*		0.831	0.007**
Dryadula phaetusa	2.964*	-0.889	-1.274	1.591		0.867
Dryas iulia	4.688***	0.419	0.286	3.506**	1.531	

Table S2. Naïve colour preference for each species included in the long-term memory assay, related to Figures 2 and S2. Results for each species for generalised linear mixed models including only ID as a random effect. P-values less than 0.05 indicate significant deviation from 50%. * = P<0.05, ** = P<0.01, *** = P<0.001.

	**z value**	**Pr(>|z|)**
H. erato	5.781	<0.0001***
H. melpomene	-0.13	0.897
H. hecale	-0.511	0.609
Agraulis vanillae	4.381	<0.0001***
Dryadula phaetusa	1.163	0.245
Dryas iulia	-0.95	0.342

Table S3. Pairwise comparisons between species in fidelity to the trained colour cue during the first recall test, related to Figure 2. Bottom left shows z-ratio and top right the associated P-value, corrected for multiple comparisons using the Šidák correction. *=P<0.05.

	H. erato	*H. melpomene*	*H. hecale*	*A. vanillae*	*D. phaetusa*	*D. iulia*
H. erato		0.909	0.529	0.975	0.022*	0.01*
H. melpomene	1.028		0.998	0.998	0.444	0.362
H. hecale	1.705	0.446		0.870	0.491	0.359
Agraulis vanillae	0.755	-0.441	-1.128		0.054	0.022*
Dryadula phaetusa	3.124*	1.833	1.762	2.820		1.000
Dryas iulia	3.364*	1.967	1.971	3.132*	0.025	

Table S4. Pairwise comparisons for within species shifts in colour preferences across trials during the long-term memory assay, related to Figure 2. Multiple comparisons corrected for using the Šidák correction. Trained = 16-hour recall after four days of training; LTM = recall after eight days feeding on neutral (white) feeders; LTM2 = recall performance after an additional four days on white feeders.

Species		Contrast	z ratio	P value
Heliconius	***H. erato***	Naïve – Trained	-10.785	<0.0001***
		Trained – LTM	3.506	0.0009***
	H. melpomene	Naïve – Trained	-9.202	<0.0001***
		Trained – LTM	2.558	0.0313*
		LTM-LTM2	0.230	0.994
	H. hecale	Naïve – Trained	-14.035	<0.0001***
		Trained – LTM	6.511	<0.0001***
		LTM-LTM2	1.470	0.367
**Non-*Heliconius***	***Agraulis vanillae***	Naïve – Trained	-16.495	<0.0001***
		Trained – LTM	11.446	<0.0001***
		LTM-LTM2	1.070	0.637
	Dryadula phaetusa	Naïve – Trained	-9.958	<0.0001***
		Trained – LTM	5.651	<0.0001***
		LTM-LTM2	1.600	0.294
	Dryas iulia	Naïve – Trained	-12.303	<0.0001***
		Trained – LTM	8.316	<0.0001***

Table S5. Pairwise comparisons between species in recall accuracy after eight days feeding on neural (white) feeders, related to Figure 2. Significance assessed using the z-test. Top right shows P-values corrected for multiple comparisons using the Šidák correction. Bottom left shows uncorrected P-values. * = P<0.05; ** = P<0.01; *** = P<0.001.

	H. erato	***H. melpomene***	***H. hecale***	***A. vanillae***	***D. phaetusa***	***D. iulia***
H. erato		1.000	0.2600	0.0002***	0.002**	<0.0001***
H. melpomene	0.9766		0.425	0.0014**	0.009**	0.0001***
H. hecale	0.0199*	0.0362*		0.258	0.662	0.018*
Agraulis vanillae	<0.0001***	0.0001***	0.0197*		1.000	0.998
Dryadula phaetusa	0.0001***	0.0006***	0.0697	0.783		0.989
Dryas iulia	<0.0001***	<0.0001***	0.0012**	0.338	0.262	

Table S6. Colour preference between purple and yellow for each species 13 days after training ended, related to Figure 2. Results for each species for generalised linear mixed models including only ID as a random effect. P-values less than 0.05 indicate significant deviation from 50% preference between purple and yellow. *** = P<0.001.

	z value	Pr(>|z|)
H. melpomene	3.745	0.00018***
H. hecale	1.824	0.0682
Agraulis vanillae	-0.593	0.553
Dryadula phaetusa	-0.82	0.412

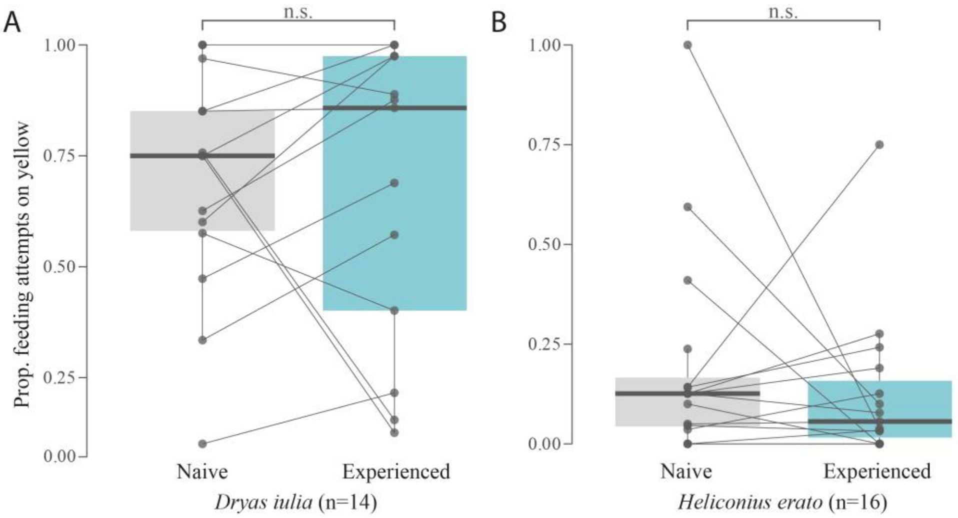

Figure S3. Control group butterflies show no significant shifts in colour preference after exposure to purple and yellow feeders, related to Figure 3. (A) Proportion of feedings attempts on yellow feeders before and after four days' exposure in Control group *Dryas iulia* and **(B)** *Heliconius erato*. Control butterflies were kept for four days in an environment where half of the purple feeders were presented with a sugar-pollen reward and half with an aversive quinine solution, likewise for yellow feeders. The boxes encompass the two middle quartiles, with central line showing median. Whiskers extend to the furthest data point within 1.5 times the interquartile range. Differences between groups were assessed using a t-test.

Table S7. Pairwise comparisons between colour preference before and after experience with coloured feeders for Control group *Dryas iulia* and *Heliconius erato*, related to Figures 3 and S3. Corrected for multiple comparisons using Tukey's test.

Species	t ratio	P value
Dryas iulia	0.587	0.916
H. erato	2.177	0.652

Table S8. Sex distributions of *Heliconius erato* and *Dryas iulia* sampled for neuroanatomy, related to Figures 3 and 4.

Group	Species	Male	Female
**Day 0**	*Dryas iulia*	6	4
	H. erato	6	7
**Learning**	*Dryas iulia*	9	14
	H. erato	16	9
**Control**	*Dryas iulia*	5	5
	H. erato	9	6

Table S9. Sex effects on the measured neuroanatomical traits in *Heliconius erato* and *Dryas iulia*, related to Figures 3 and 4. Effects tested using generalised linear models with Gaussian distributions.

Trait	Species	χ^2	Df	Pr(>χ^2)
**Calyx synapse density**	*Dryas iulia*	1.134	1	0.287
	H. erato	0.179	1	0.672
**Mushroom body calyx volume**	*Dryas iulia*	1.870	1	0.172
	H. erato	4.502	1	0.034*
**Number of calyx synapses**	*Dryas iulia*	0.469	1	0.494
	H. erato	0.423	1	0.515
**Number of Kenyon cells**	*Dryas iulia*	0.014	1	0.907
	H. erato	4.963	1	0.026*

Table S10. Species and group effects on mushroom body development in *Heliconius erato* and *Dryas iulia*, related to Figure 3. ANOVA results from a series of GLMs testing for variation between *Dryas iulia* and *Heliconius erato*, divided into three treatment groups – Day 0, Learning and Control – in several neuroanatomical traits measured in the mushroom body calyx. Sex was found to have a significant effect only on calyx volume in *Heliconius erato* and was therefore included as a random effect when testing for differences in that trait only.

Trait	Factor	χ^2	Df	Pr(>χ^2)
**Calyx synapse density**	**Group**	33.771	2	<0.0001***
	**Species**	1.373	1	0.241
	**Group:Species**	6.312	2	0.0426*
**Mushroom body calyx volume**	**Group**	32.729	2	<0.0001***
	**Species**	817.809	1	<0.0001***
	**Group:Species**	5.355	2	0.068
**Number of calyx synapses**	**Group**	25.857	2	<0.0001***
	**Species**	40.056	1	<0.0001***
	**Group:Species**	10.338	2	0.0057**
**Number of Kenyon cells**	**Group**	4.522	2	0.104
	**Species**	770.822	1	<0.0001***
	**Group:Species**	0.160	2	0.923

Table S11. Selected pairwise comparisons in several neuroanatomical traits measured in the mushroom body calyx, related to Figure 3. Comparisons are made within species between groups, and between species for individuals in the same treatment group. Corrected for multiple comparisons using the Šidák test.

Trait	Species	Contrast	t ratio	Uncorrected P value	Corrected P value
**Calyx synapse density**	***Dryas iulia***	Day 0 – Learning	1.265	0.209	0.505
		Day 0 – Control	1.888	0.062	0.176
		Learning – Control	0.932	0.354	0.730
	H. erato	Day 0 – Learning	4.490	<0.0001***	<0.0001***
		Day 0 – Control	5.916	<0.0001***	<0.0001***
		Learning – Control	2.163	0.033*	0.097
	Dryas iulia* – *H. erato	Day 0	-1.519	0.132	0.347
		Learning	1.387	0.169	0.426
		Control	1.859	0.066	0.186
**Mushroom body calyx volume**	***Dryas iulia***	Day 0 – Learning	-2.264	0.027*	0.077
		Day 0 – Control	-1.396	0.318	0.318
		Learning – Control	0.346	0.960	0.960
	H. erato	Day 0 – Learning	-5.384	<0.0001***	<0.0001***
		Day 0 – Control	-4.506	<0.0001***	<0.0001***
		Learning – Control	0.899	0.372	0.752
	Dryas iulia* – *H. erato	Day 0	-13.096	<0.0001***	<0.0001***
		Learning	-19.732	<0.0001***	<0.0001***
		Control	-16.136	<0.0001***	<0.0001***
**Number of calyx synapses**	***Dryas iulia***	Day 0 – Learning	0.429	0.669	0.964
		Day 0 – Control	0.973	0.334	0.704
		Learning – Control	0.694	0.490	0.867
	H. erato	Day 0 – Learning	3.519	0.0007***	0.0022**
		Day 0 – Control	5.928	<0.0001***	<0.0001***
		Learning – Control	2.700	0.0064**	0.0254*
	Dryas iulia* – *H. erato	Day 0	-5.783	<0.0001***	<0.0001***
		Learning	-3.872	0.0002	0.0007***
		Control	-1.399	0.166	0.420
**Number of Kenyon cells**	***Dryas iulia***	Day 0 – Learning	-1.470	0.146	0.377
		Day 0 – Control	-1.460	0.149	0.383
		Learning – Control	-0.198	0.844	0.996
	H. erato	Day 0 – Learning	-0.880	0.382	0.764
		Day 0 – Control	-1.373	0.174	0.436
		Learning – Control	-0.497	0.621	0.945
	Dryas iulia* – *H. erato	Day 0	-15.153	<0.0001***	<0.0001***
		Learning	-17.280	<0.0001***	<0.0001***
		Control	-15.417	<0.0001***	<0.0001***

Table S12. Group and species variations in the scaling relationships of the mushroom body, related to Figure 3. Pairwise comparisons for changes in elevation in the scaling relationship between Kenyon cell number and synapse number in the calyx, and Kenyon cell number and calyx volume, related to Figure 3.

Regression	Species	Contrast	Test stat	Uncorrected P value	Corrected P value
**Number of calyx synapses ~ Number of Kenyon cells**	***Dryas iulia***	Day 0 – Learning	2.384	0.123	0.859
		Day 0 – Control	5.111	0.0237*	0.303
		Learning – Control	0.568	0.451	0.999
	H. erato	Day 0 – Learning	7.431	0.0064**	0.092
		Day 0 – Control	24.969	<0.0001***	<0.0001***
		Learning – Control	5.268	0.0217*	0.327
	Dryas iulia* – *H. erato	Day 0	4.866	0.0274*	0.341
		Learning	26.805	<0.0001***	<0.0001***
		Control	18.126	<0.0001***	0.0003***
**Calyx volume ~ Number of Kenyon cells**	***Dryas iulia***	Day 0 – Learning	0.526	0.468	0.999
		Day 0 – Control	0.0003	0.986	1.000
		Learning – Control	0.0300	0.862	1.000
	H. erato	Day 0 – Learning	9.727	0.0018**	0.0269*
		Day 0 – Control	5.489	0.0191*	0.252
		Learning – Control	2.557	0.110	0.825
	Dryas iulia* – *H. erato	Day 0	0.625	0.429	0.999
		Learning	0.00288	0.957	1.000
		Control	0.618	0.432	1.000

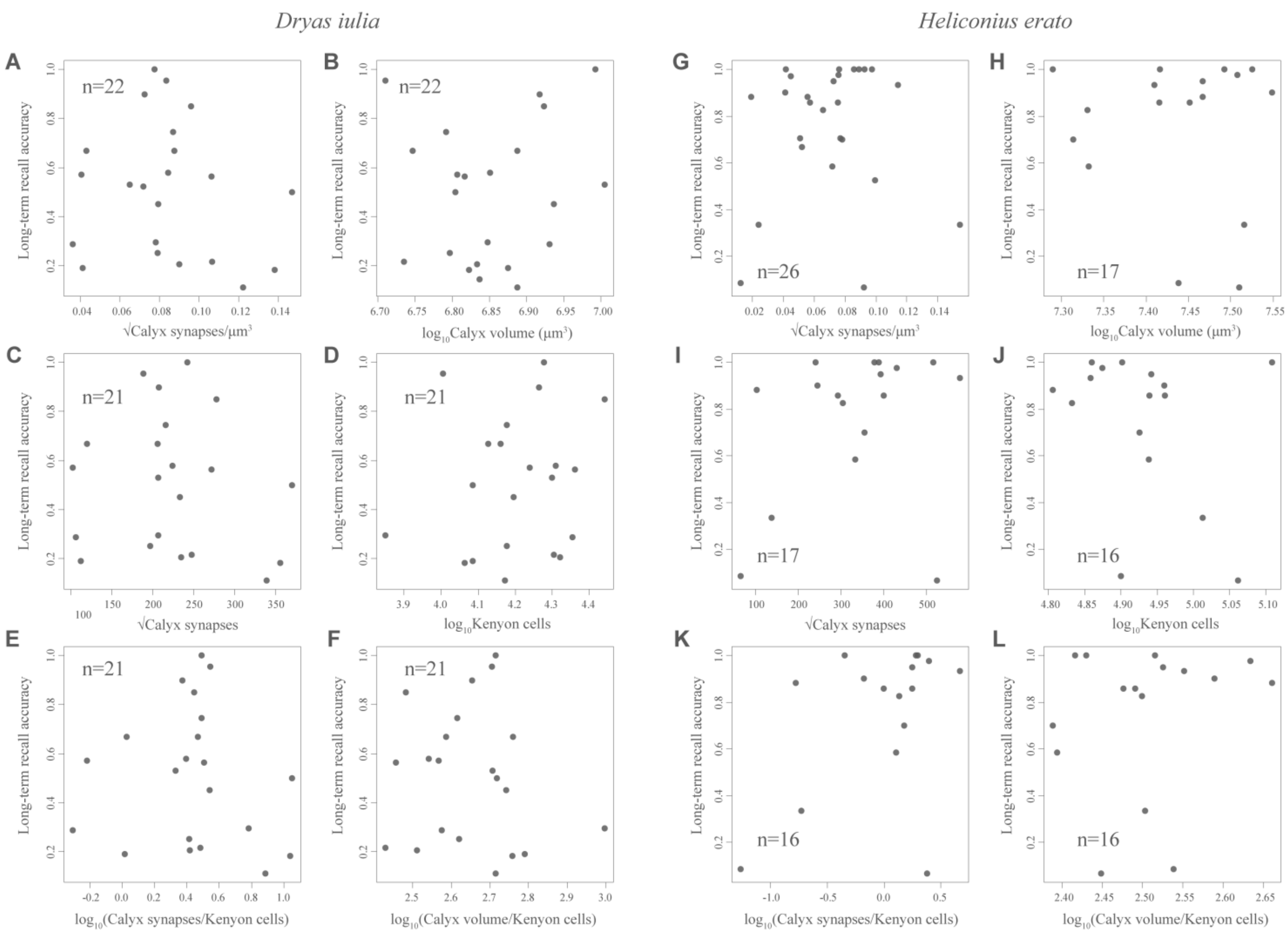

Figure S4. Individual performance in the long-term recall test (after eight days fed on neutral (white) feeders) is not correlated with any of the traits measured in the mushroom body calyx for either *Dryas iulia* or *Heliconius erato*, related to Figure 4. Relationships were tested using generalised linear mixed models, with significance assessed using a chi-square test. All values correspond to a single hemisphere of the brain.

Table S13. Relationships between recall performance and measured neuroanatomical traits in *Dryas iulia* and *Heliconius erato*, related to Figures 4 and S4. ANOVA results for a series of GLMMs testing for relationships between neuroanatomical measurements in the mushroom body calyx and performance in the initial and long-term recall tests in *Dryas iulia* and *Heliconius erato*.

Trait	Test	Species	χ^2	Df	Pr(>χ^2)
**Synapse density**	**Initial recall**	***Dryas iulia***	1.4775	1	0.224
		H. erato	5.473	1	0.0193*
	**Long-term recall**	***Dryas iulia***	0.4562	1	0.499
		H. erato	0.1482	1	0.700
**Mushroom body calyx volume**	**Initial recall**	***Dryas iulia***	7.6747	1	0.0056**
		H. erato	0.676	1	0.411
	**Long-term recall**	***Dryas iulia***	0.0368	1	0.848
		H. erato	0.099	1	0.753
**Number of calyx synapses**	**Initial recall**	***Dryas iulia***	0.3431	1	0.558
		H. erato	9.199	1	0.0024**
	**Long-term recall**	***Dryas iulia***	0.3575	1	0.550
		H. erato	1.585	1	0.208
**Number of Kenyon cells**	**Initial recall**	***Dryas iulia***	0.0068	1	0.934
		H. erato	1.585	1	0.208
	**Long-term recall**	***Dryas iulia***	0.4704	1	0.493
		H. erato	1.5167	1	0.218
**Ratio of synapses to Kenyon cells**	**Initial recall**	***Dryas iulia***	0.576	1	0.448
		H. erato	12.852	1	0.00034***
	**Long-term recall**	***Dryas iulia***	0.2323	1	0.630
		H. erato	3.2646	1	0.0708
**Ratio of calyx volume to Kenyon cells**	**Initial recall**	***Dryas iulia***	1.8266	1	0.177
		H. erato	0.2405	1	0.624
	**Long-term recall**	***Dryas iulia***	0.3875	1	0.534
		H. erato	0.3793	1	0.538

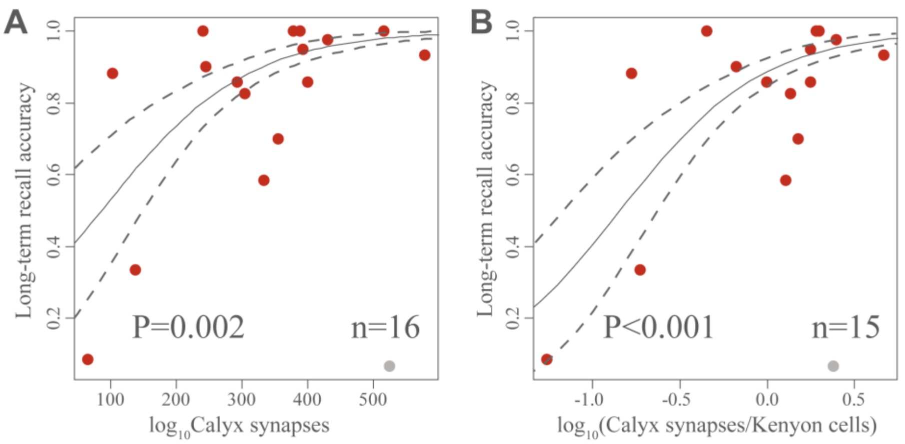

Figure S5. Long-term recall (8 days) accuracy and synapse number in *Heliconius erato*, related to Figures 4 and S4. When a single outlier individual (E43, grey) is removed, **(A)** the total synapse number in the calyx (χ^2=9.263, d.f.=1, P=0.002) and **(B)** the ratio of synapses to Kenyon cells (χ^2=10.918, d.f.=1, P=0.001) show a significant positive relationship with performance in the long-term recall test. This individual showed unusual behaviour, making 16 out of 16 correct feeding attempts in the initial recall test, but only 2 out of 28 correct attempts in the long-term recall test. Regression lines, with standard errors, are from generalised linear mixed models, assessed using a chi-square test. All values correspond to a single hemisphere of the brain.

Table S14. Removing E43 has an outsized effect on the results compared with removing other individuals, related to Figures 4, S4 and S5. Table shows results from a series of generalised linear mixed models where individuals were excluded from the dataset one at a time. Asterisks indicate significant pairwise differences. * = P<0.05, ** = P<0.01, *** = P<0.001.

ID removed	Synapse count χ^2	Synapse count P	Synapse/KC ratio χ^2	Synapse/KC ratio P
E09	2.042	0.153	4.278	0.039*
E10	1.520	0.218	3.044	0.081
E13	0.122	0.727	0.389	0.533
E16	1.405	0.236	2.688	0.101
E18	2.190	0.139	4.371	0.037*
E19	1.560	0.212	3.372	0.066
E22	1.382	0.240	2.780	0.095
E27	1.232	0.267	2.552	0.110
E29	0.741	0.389	1.929	0.165
E30	0.985	0.321	-	-
E31	1.402	0.236	2.82	0.093
E34	1.495	0.221	3.074	0.080
E37	1.374	0.241	2.784	0.095
E41	1.654	0.199	3.210	0.073
E43	9.263	0.002***	10.918	<0.001***
E46	1.530	0.216	3.045	0.081
E47	1.584	0.208	3.280	0.070

Table S15. Removing E43 produces stable results. After removing individual E43, we then re-analysed the data removing other individuals one at a time to test whether removing E43 produces stable results. The table shows that removing E43 from the dataset produces stable results, even when removing other individuals. Asterisks indicate significant pairwise differences. * = P<0.05, ** = P<0.01, *** = P<0.001.

ID	Synapse count		Synapse/KC ratio	
removed	χ^2	P	χ^2	P
E09	13.29	<0.001***	15.491	<0.001***
E10	8.899	0.003***	10.421	0.001***
E13	4.025	0.045*	3.394	0.065
E16	9.164	0.002***	10.279	0.001***
E18	11.358	<0.001***	13.739	<0.001***
E19	10.505	0.001***	14.079	<0.001***
E22	9.918	0.002***	10.060	0.002***
E27	7.775	0.005***	9.181	0.002***
E29	6.090	0.014*	7.209	0.007***
E30	8.091	0.004***	-	-
E31	8.112	0.004***	9.547	0.002***
E34	8.419	0.004***	10.435	0.001***
E37	8.686	0.003***	10.167	0.001***
E41	9.264	0.002***	10.549	0.001***
E46	8.586	0.003***	10.078	0.002***
E47	9.510	0.002***	11.604	<0.001***
